# Effect of Dietary Components on Larval Life History Characteristics in the Medfly (*Ceratitis capitata*: Diptera, Tephritidae)

**DOI:** 10.1371/journal.pone.0086029

**Published:** 2014-01-21

**Authors:** William J. Nash, Tracey Chapman

**Affiliations:** School of Biological Sciences, University of East Anglia, Norwich Research Park, Norwich, Norfolk, United Kingdom; University of Arkansas, United States of America

## Abstract

**Background:**

The ability to respond to heterogenous nutritional resources is an important factor in the adaptive radiation of insects such as the highly polyphagous Medfly. Here we examined the breadth of the Medfly’s capacity to respond to different developmental conditions, by experimentally altering diet components as a proxy for host quality and novelty.

**Methodology/Principal Findings:**

We tested responses of larval life history to diets containing protein and carbohydrate components found in and outside the natural host range of this species. A 40% reduction in the quantity of protein caused a significant increase in egg to adult mortality by 26.5%±6% in comparison to the standard baseline diet. Proteins and carbohydrates had differential effects on larval versus pupal development and survival. Addition of a novel protein source, casein (i.e. milk protein), to the diet increased larval mortality by 19.4%±3% and also lengthened the duration of larval development by 1.93±0.5 days in comparison to the standard diet. Alteration of dietary carbohydrate, by replacing the baseline starch with simple sugars, increased mortality specifically within the pupal stage (by 28.2%±8% and 26.2%±9% for glucose and maltose diets, respectively). Development in the presence of the novel carbohydrate lactose (milk sugar) was successful, though on this diet there was a decrease of 29.8±1.6 µg in mean pupal weight in comparison to pupae reared on the baseline diet.

**Conclusions:**

The results confirm that laboratory reared Medfly retain the ability to survive development through a wide range of fluctuations in the nutritional environment. We highlight new facets of the responses of different stages of holometabolous life histories to key dietary components. The results are relevant to colonisation scenarios and key to the biology of this highly invasive species.

## Introduction

The nutrients that an organism absorbs from its diet are essential for development, and determine how organisms can maximise their fitness [1], [2]. In holometabolous insects, alteration in diet quality during development has wide ranging effects upon many life history characteristics [3]. The two major nutritive components of diet that contribute to development are proteins and carbohydrates. Proteins provide essential amino acids necessary for viability. Imbalances in dietary amino acids can have significant effects upon development and fitness [4] and may underlie the effect of dietary restriction on lifespan [5]. Carbohydrates provide energy to fuel development and represent the mechanism by which energy is stored for future use [4]. The availability of different nutrients during the developmental phase determines characteristics such as growth rate [6], developmental survival and also impacts upon adult traits such as body size [7].

The relationship between dietary macronutrients and the development of traits in heterogeneous nutritional environments can be described by the geometric framework (GF) [8]–[10]. In considering an individual’s nutritional environment as a multidimensional space with individual nutrients as its axes, the GF establishes the optimal nutritional state for that individual as a ‘nutritional target’. Within the GF, the trajectory at which an individual moves through nutritional space is referred to as a ‘nutritional rail’. In an environment where diet components occur in a fixed ratio, progress towards the nutritional target is made along a single rail. However, if the ratio of nutrients in the environment is imbalanced or varies, progress towards the target is achieved by altering the intake of different nutrient components [8].

Holometabolous insects maintain robust mechanisms to ensure that development is successful in the environment in which their larvae develop, and hence that the nutritional target is obtained. The interaction between larval growth rate, critical weight and the endocrinological control of larval development offers the possibility of significant plasticity in the determination of adult size and energy stores [11]–[15]. Critical weight is a point reached during the exponential growth rate of the final larval instar, which determines when the process of pupation can begin. This critical weight is influenced by diet quality and is relatively insensitive to external environmental factors [12], [13]. Critical weight thus allows an insect to adapt the rate of its development to diverse nutritional environments in order to optimise key adult traits, such as body size [16].

The present study focuses on a highly successful generalist species and its ability to adapt its developmental life history to changes in specific nutrients within the larval environment. The Mediterranean fruit fly (Medfly), *Ceratitis capitata*, is highly polyphagous, infesting over 350 hosts [17] and can successfully utilise oviposition sites beyond its natural host range [18]. Experimental evidence has revealed that the Medfly can show considerable flexibility in its life history, permitting the use of a diverse range of larval diets [19]–[24]. These findings suggest striking variability in the genes underlying diet selection and utilisation. Indeed, following artificial selection, Medfly can even be reared successfully on diets derived entirely from a non-herbivorous source [25].

The Medfly is a globally important agricultural pest, and effective mass rearing strategies have been developed as part of sterile insect technique (SIT) programmes [26]. These have highlighted the importance of the larval diet in determining adult mating success, and show that adults reared on poor diets suffer reduced fitness [24], [27]–[29]. Protein deficiency in the larval environment also reduces body size in wild [18], [29] and laboratory [27] populations. This is important as small body size is associated with reduced male mating success [27], [30]. Reduced protein can also delay larval development and reduce survival to adult eclosion [24], [31], [32]. Large, protein fed males are more likely to have their sperm stored in the female and to have more sperm stored [33]. Dietary effects on body size could be mediated through alterations in the quantity of nutrients stored as lipids and as proteins prior to pupariation [23], [24]. In females, the nutritional quality of larval diet affects ovarian development and egg production [34]–[36].

An important omission from existing studies of diet on development, however, is the effect of nutrient quality (via use of existing and novel hosts) as well as quantity on different developmental stages. This is relevant to our understanding how the Medfly can tailor its developmental progress towards a nutritional target, as well as for further development of husbandry in SIT programmes. We addressed this omission by testing the effect of standard and novel protein and carbohydrate components on the developmental life history of Medflies. We altered diet components to provide variation in both host quality and host novelty, using protein and carbohydrate sources both inside and outside the natural host range.

## Materials and Methods

### Origin and Maintenance of Fly Stocks

The study was conducted using *Ceratitis capitata* from the Toliman wild type strain, sourced from the Guatemalan Mass rearing facility, and raised under laboratory conditions since 1990 [37]. Prior to experimentation flies were reared in 1L cages with 50 individuals per cage at an approximate 1∶1 sex ratio. Adult flies were fed a 3∶1 sucrose:yeast hydrolysate diet and water *ad libitum*. Cages were maintained on a 12∶12 light dark photoperiod at 25°C and experiments were also conducted under these same conditions. Stock lines were reared on a bran-based larval diet (Brewer’s Yeast 147.3 g/L, Sucrose 295 g/L, Citric Acid 10.1 g/L, Sodium benzoate 9.1 g/L, Wheat 440 g/L, Water 1L). Eight generations prior to the experiments, stock populations were placed on a starch-based larval diet (Agar 5 g/L, Starch 30 g/L, Brewer’s Yeast 30 g/L, Propionic Acid 5 ml/L, Water 1L). Each generation, approximately 500 eggs were placed on 100 ml of starch diet in a glass bottle. When 3^rd^ Instar larvae started to ‘jump’ from the larval medium, the bottles were laid on sand and pupae allowed to emerge for 7 days.

### Dietary Treatments

Wild type flies from the Toliman mass rearing strain were used in these experiments (see Supplementary Methods for details). Six diet treatments were used in addition to the standard starch larval diet upon which the flies were maintained (Table 1). The first three larval diets altered protein sources. The ‘High Protein’ diet contained 40% more protein (yeast hydrolysate) than the standard starch larval diet, ‘Low Protein’ contained 40% less yeast and the ‘casein’ diet replaced the yeast with an equal quantity of casein, one of the two main proteins in cow’s milk. We supplemented the casein diet with multivitamin powder (Boots) and table salt (Saxa) (Table 1) to compensate for non-protein differences in comparison to the high/low protein diets. The second set of three diets altered carbohydrate sources. In the ‘glucose’ diet, the polysaccharide of the starch diet was substituted by an equal quantity of its monosaccharide glucose base, in the ‘maltose’ diet starch was replaced by the disaccharide sugar maltose (two glucose molecules joined by α(1→4) bond; [38]); and finally in the ‘lactose’ treatment, the starch was replaced by the disaccharide milk sugar lactose (glucose+galactose joined by β(1→4) bond; [39]). Note that these carbohydrate diets contained a standard quantity of yeast (50 g/L), which itself contains other sources of carbohydrate in small amounts. Therefore only the major carbohydrate source was varied in these diets.

**Table 1:** Larval diets used in this study.

Ingredient	Starch	High Protein	Low Protein	Casein (Milk Protein)	Glucose	Maltose	Lactose	
Water	1000	1000	1000	1000	1000	1000	1000	ml
Agar	15	15	15	15	15	15	15	g
Starch	30	30	30	30	–	–	–	g
Glucose	–	–	–	–	30	–	–	g
Maltose	–	–	–	–	–	30	–	g
Lactose	–	–	–	–	–	–	30	g
Yeast	50	70	30	–	50	50	50	g
Casein	–	–	–	50	-	–	–	g
Propionic Acid	5	5	5	–	5	5	5	ml
Multivitamin powder	–	–	–	0.3	–	–	–	g
Salt	–	–	–	0.3	–	–	–	g
Nipagin	–	–	–	25	–	–	–	ml

### Experimental Protocol

The experiment was conducted in two blocks, over consecutive generations, first the protein then the carbohydrate experiment. For each replicate 100 eggs were selected at random using a light microscope and placed on 55 mm disc of Whatman filter paper soaked in dH_2_O. This disk was then placed on 20 g of the appropriate diet in a Petri dish. This process was repeated for each dietary treatment in a block, as well as for 20 g of the standard Starch diet, which acted as a baseline control for each block of the experiment. Egg samples were allocated at random to the diet treatments. The four Petri dishes within each block were then treated as one replicate for each diet. Five replicates were conducted for the protein experiment (n = 5 replicates of 100 eggs for each of the 4 diet treatments), and six replicates for the carbohydrate experiment (n = 6 replicates of 100 eggs for each of the 4 treatments). Petri dishes were sealed using ‘Parafilm’ for 11 days, at which point the film was removed and the plates placed in larger boxes to allow larvae access to sand for pupation. Larvae were allowed 5 days, following the emergence of the first pupae, to exit the larval diet to pupate. It was uncommon for larvae to remain in the food at the end of this period, and any remaining larvae at this point were discarded from the experiment.

### Larval and Pupal Development Time

The number of pupae emerging each day was recorded on each of the 5 days allowed for pupation, allowing calculation of mean larval development time. The daily cohort of emerging pupae was sieved from the sand and transferred to a Petri dish. These Petri dishes were then checked daily for adult emergence. Adults were counted and their sex recorded, allowing mean pupal development time to be calculated for each replicate. Individuals that only partially emerged were discarded from the experiment. Overall, development time was calculated by summing the mean larval and pupal development time of each replicate.

### Larval and Pupal Survival

The total number of pupae present at the end of the 5 days allowed for pupation was recorded as a measure of larval survival. The total number of fully emerging adults was used as the measure of pupal survival. Overall survival for each replicate was calculated by subtracting the number of surviving adults from the original replicate population size of 100 eggs.

### Pupal Weight

Each cohort of pupae were weighed on the day of emergence, and the total weight of the cohort divided by the number of pupae per cohort in order to give the mean pupal weight per treatment per day. Pupal weight was used as a proxy for adult size.

### Data Analysis

Data analysis was conducted in R v2.13.2 [40]. The data for the protein and carbohydrate experiments were analysed separately. Development time was measured as a count of the number of days between each developmental period and analysed by generalised linear model (GLM) using the Poisson distribution. Survival was treated as proportion data (proportion of individuals entering the developmental stage that successfully completed it) and analysed by GLMs using the binomial distribution. Covariance between the proportion of surviving individuals and experimental day was analysed by GLM ANCOVA. Weight data were analysed using ANOVA, and GLM ANCOVA to incorporate emergence day. Data that were overdispersed were analysed using quasipoisson and quasibinomial distributions. Binomial data that displayed heteroscedasticity were weighted according to the inverse proportion of the dispersion of the data. After each model was fitted, significance of treatment comparisons was assessed using Tukey HSD multiple comparison tests (‘multcomp’ package; [41]) in R. Bonferroni corrections were applied to the results to control for multiple comparisons.

## Results

### 1. Egg to Adult Survival

**Figure 1:**
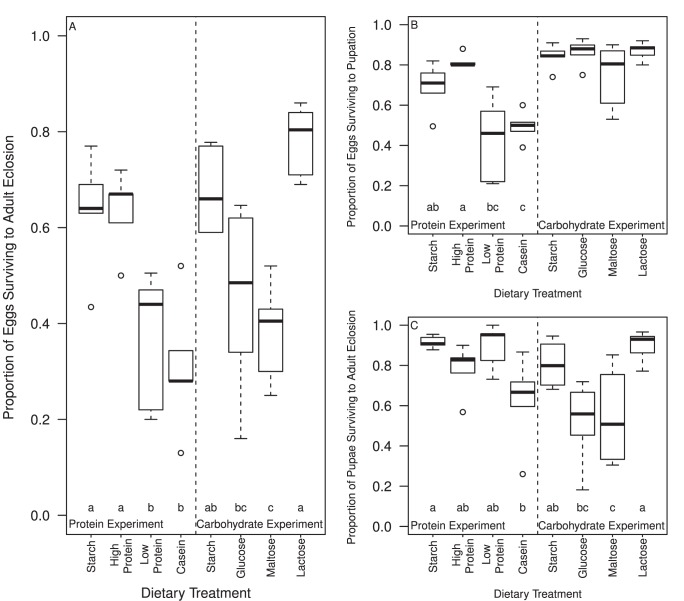
Proportion of Medfly individuals surviving between each developmental stage when reared upon different dietary treatments. For each panel, on the left are the 4 diets with altered protein content and on the right the 4 diets with altered carbohydrate content. (a) Overall proportion of eggs surviving from egg laying to adult eclosion. (b) Proportion of eggs surviving to pupal formation. (c) Proportion of pupae surviving from initial pupal formation to adult eclosion. Dotted lines represent the range of the data; outer limits of the boxes indicate inter quartile range and the black line at the centre of each box represents the median value. Circles represent outliers. Letters indicate groupings significantly different following post hoc tests (see text for details).

The number of individuals surviving from egg to adult was significantly altered by both the protein and carbohydrate diet manipulations. There was an overall effect of protein treatment (F_3,16_ = 7.878, *P* = 0.002; Fig. 1a). A significantly lower proportion of eggs reared on low protein survived to adulthood than did those reared on starch (*P* = 0.008) and high protein diets (*P* = 0.01). Also, a significantly lower proportion of eggs reared on the casein diet survived to adulthood than those reared on starch (*P* = 0.002) and high protein diets (*P* = 0.003). There was also a significant effect of carbohydrate treatment on overall egg to adult survival (F_3,20_ = 13.962, *P*<0.001; Fig. 1a). A significantly lower proportion of eggs reared on the glucose diet survived to adult eclosion than did those reared on lactose (*P*<0.001) and starch (*P* = 0.02). Survival was also lower on the maltose in comparison to the lactose (*P*<0.001) and starch (*P*<0.001) diets.

#### 1.1 Larval survival

Protein treatment had a significant effect on larval survival, i.e. the number of individuals surviving from egg to pupae (F_3,16_ = 10.742, P<0.001; Fig. 1b). A significantly lower proportion of larvae reared on low protein and casein diets survived to pupation than those reared on the high protein diet (post hoc tests, P<0.001). Larval survival was also significantly lower on the casein in comparison to starch-based diet (P = 0.028). In contrast, variation in carbohydrates had no significant effect on larval survival (F_3,20_ = 1.8253, P = 0.175; Fig. 1b).

#### 1.2 Pupal survival

Pupal survival (i.e. the proportion of pupae eclosing as adults) was also significantly altered by protein treatment (F_3, 16_ = 3.6825, P = 0.03; Fig. 1c). The proportion of pupae surviving to adult eclosion was significantly lower for the casein in comparison to the starch diet (post hoc tests, P = 0.02). In contrast to larval survival, carbohydrate treatment did have a significant effect on pupal survival (F_3,20_ = 9.1262, P<0.001; Fig. 1c). A significantly lower proportion of pupae reared on the glucose diet survived to adulthood than did those raised on lactose (P<0.001). Also, the proportion of pupae surviving to adult eclosion on the maltose diet was significantly lower than for pupae reared on lactose (P<0.001) and starch (P = 0.03).

Overall, diet components had contrasting effects on survival through the different life history stages of development, with a large effect of protein on survival during the larval growth phase and of carbohydrate on survival during the pupal phase.

### 2. Development Time

**Figure 2:**
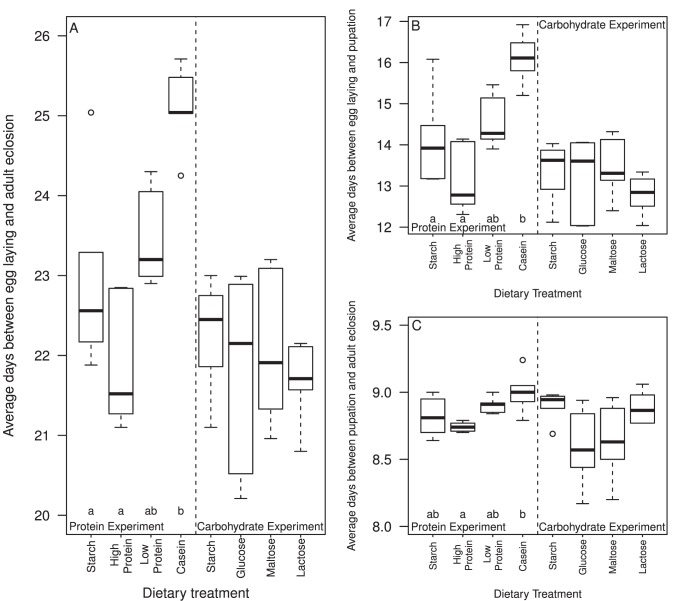
Average duration of each stage of development of Medfly reared upon different dietary treatments. For each panel, the protein experiment is on the left hand side, carbohydrate experiment on the right hand side. (a) Average duration of overall development (median days) from egg laying to adult eclosion. (b) Average duration of development (median days) of the larval stage, from egg to pupal formation. (c) Average duration of the pupal stage (median days) from pupal formation to adult eclosion. Boxplots are as defined in Fig. 1.

Development time was significantly altered by protein treatments (F_3,16_ = 11.548, *P*<0.001; Fig. 2a). Eggs reared on casein took significantly longer to develop to adulthood than did eggs reared on starch (post hoc tests, *P* = 0.009) and high protein (*P*<0.001). Interestingly, carbohydrate treatment had no significant effect on the overall duration of development (F_3,20_ = 0.5405, *P* = 0.660; Fig. 2a).

#### 2.1 Larval development time

Protein had a significant effect on larval development (i.e. duration of development from egg to pupa; F_3,16_ = 9.5858, P<0.001; Fig. 2b). The development time of larvae reared on casein was significantly longer than for starch (post hoc tests, P = 0.018) and high protein (P<0.001). Carbohydrate treatment had no significant effect on the mean duration of larval development (F_3,20_ = 0.9082, P = 0.455; Fig. 2b).

#### 2.2 pupal development time

Protein treatment had a significant effect on pupal development (time from pupa to adult eclosion; F_3,16_ = 4.3837, P = 0.02; Fig. 2c). The development time of pupae reared on casein was significantly longer than that of pupae reared on high protein (post hoc tests, P = 0.02). Carbohydrate treatment had a marginally significant effect on the duration of pupal development (F_3,20_ = 3.5694, P = 0.032; Fig. 2c). However, this effect was non significant following post hoc tests.

### 3. Rate of Development

#### 3.1 Pupal emergence

To analyse differences in the rate at which pupae were formed across each diet treatment (i.e. the number of pupae which were formed each day over 5 days), we fitted a GLM ANCOVA model including experimental day as a covariate. There was no significant interaction between protein diet treatment and day (F_3,89_ = 0.007, P = 0.999) and this term was removed from the model. The resulting analysis revealed significant effects of protein treatment (F_3,93_ = 20.015, P<0.001) and experimental day (F_1,92_ = 75.555, P<0.001) on the proportion of pupae formed per day (Fig. S1a). The mean proportion of larvae forming pupae each day was significantly different across all diets (post hoc tests, P≤0.001) with the exception of the low protein and casein-based diets, which were not significantly different from one another (P = 0.4). In the analyses of carbohydrates, we found that the interaction between carbohydrate and experimental day was also non significant (F_3,95_ = 1.3509, P = 0.263) and it was therefore removed. The resulting analysis revealed no effect of carbohydrate on the proportion of pupae forming per day (F_3, 99_ = 2.487, P = 0.07), but a significant effect of experimental day (F_1,98_ = 32.785, P<0.001; Fig. S1b).

#### 3.2 Adult eclosion rate

We tested for differences in the rate of adult emergence (the number of adults eclosing per day across the different diets) using a GLM ANCOVA model. There was no significant interaction between protein diet and day (F_3,99_ = 1.4726, P = 0.227). With this term removed from the model, we found that protein treatment had no significant effect upon the proportion of adults eclosing per day (F_3,103_ = 1.3341, P = 0.268), though there was an effect of experimental day (F_1,102_ = 93.5152, P<0.001; Fig. S1c). In a separate model to analyse the effect of carbohydrate, the interaction between carbohydrate diet treatment and experimental day was significant (F_3,103_ = 10.727, P<0.001). There were also significant effects of carbohydrate (F_3,107_ = 8.190, P<0.001) and of experimental day (F_1,107_ = 101.287, P<0.001) on the proportion of pupae eclosing as adults (Fig. S1d). All treatments had significantly different intercepts and gradients (Table S1).

### 4. Pupal Weight

**Figure 3:**
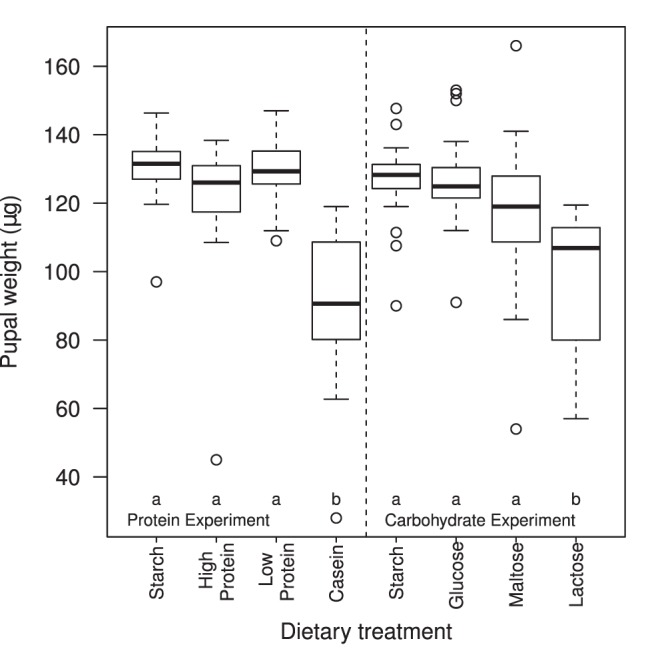
Pupal weight (median weight in µg) of Medfly following rearing upon different dietary treatments. The protein experiment is on the left hand side, carbohydrate experiment on the right hand side. Boxplot is as defined in Fig. 1.

Pupal weight was significantly affected by protein (F_3,94_ = 35.218, *P*<0.001; Fig. 3) and was significantly lower for casein than all other diets (post hoc tests, *P*<0.001, all comparisons). Carbohydrate also had a significant effect on pupal weight (F_3,93_ = 14.162, *P*<0.001; Fig. 3). Pupae reared on lactose had significantly lower mean weights than all other treatments (starch and lactose based diets *P*<0.001; maltose *P* = 0.015). To further analyse this finding, an ANCOVA, which considered pupal emergence day as a covariate of mean pupal weight, was fitted. This showed a significant interaction between protein and day of pupal emergence (F_3,90_ = 8.2832, *P*<0.001; Fig. S2a). Both diet (F_3,90_ = 48.2115, *P*<0.001), and pupal emergence day (F_1,90_ = 13.8325, *P*<0.001) also had a significant effect upon mean pupal weight. The significant interaction was driven by the negative gradient in the casein treatment (−11.28, t = −4.401, *P*<0.001). The same analysis performed for the carbohydrate experiment revealed a significant effect of diet (F_3,89_ = 15.7155, *P*<0.001) but not day (F_1 89_ = 2.4118, *P* = 0.124) on pupal weight. The interaction between diet and emergence day was significant (F_3,89_ = 3.9304, *P* = 0.011; Fig. S2b), and was driven by the interaction between the lactose and emergence day, for which the gradient was significantly negative (−11.29, t = −2.719, *P* = 0.008).

## Discussion

The results reveal that different dietary nutrients had significant but divergent effects on different stages of development in the Medfly. Decreases in protein quantity and quality had pronounced effects on larval development, increasing mortality and the duration of development. Alteration of carbohydrate quality affected mortality within the pupal stage. The results confirm that the Medfly can develop successfully on a wide range of different protein and carbohydrate sources. As a generalist, the Medfly therefore retains sufficient genetic variation to allow the expression of adaptive plasticity across a range of developmental environments [42]. This plasticity can buffer the adult phenotype against the effects of environmental variation during larval development. Such plasticity is thought to be advantageous to generalist species during colonisation, but may also have negative effects on overall adaptive radiation [43].

Our study highlights the potential importance of the holometabolous lifestyle to generalist species. The larval phase of development, where 90% of adult body mass is accrued [3] represents the defined growth phase. This allows the duration of development to be tailored to optimise progress along a nutritional rail towards the nutritional target [8], [9] to achieve a stable adult phenotype. This suggests that protein is the key nutrient during the larval phase, as duration of development increased in larvae reared on diets with reduced protein content or quality. However, individuals reared on a low protein diet did not show reduced adult body size. This trade-off between development time and body size is consistent with the endocrinological control model of holometabolous development [13]. Also, decreased numbers of individuals survived on diets with reduced protein. This shows protein to be a limiting resource during the larval phase. These results are in agreement with previous studies that manipulated protein in order to optimise the mass-rearing process [23], [24].

Individuals that successfully completed the larval growth phase and entered the metamorphic pupal phase were not affected, in the traits assayed here, by the protein content of their diets. Indeed, it was during the metamorphic phase that the effects of the carbohydrate components of diet became apparent. Larvae reared on diets containing simple carbohydrates (glucose, maltose) exhibited lower survival during metamorphosis. No effects of those carbohydrates were seen during larval development, suggesting that glucose and maltose are less efficient energy sources, or are less able to facilitate the provision of storable energy, e.g. as lipids in the fat body or as glycogen [44], [45]. If energy stores such as in the fat body can be influenced significantly by carbohydrate quality [23], [24], it will interesting to consider the wider effects this may have on adult phenotype beyond body size - for example on early life reproductive potential [46]. However, variation in stored lipids and proteins in larvae about to pupate can potentially be compensated for during metamorphosis [23]. The lack of effects of carbohydrates on growth rate or development time during the larval phase suggests that the larvae have a limited ability to compensate for poor quality carbohydrates in the diet by, for example, slowing growth rate in order to maximise carbohydrate energy storage for future development. The effects of the two major diet components protein and carbohydrate are therefore relatively independent of one another.

The only treatments that significantly altered adult size were the novel diets that fell outside of the Medfly’s natural host range (casein, lactose). Such diets can be used to simulate encounters with ‘alien’ hosts, for example during colonisation events. For a highly invasive, generalist species such as the medfly [47], which can exhibit great plasticity in the degree of host oviposition preference [48]–[51], the ability to maximise developmental success in ‘alien’ hosts is predicted to be an important trait. In our study, Medflies developed successfully on both novel host treatments, though adult body size was decreased. When the protein source was novel, all elements of development were compromised. Novel carbohydrate, on the other hand, caused no significant changes to the developmental traits assayed. However, more individuals survived than on the baseline diet and surviving pupae eclosed as adults at a faster rate.

The novel protein diet exhibited the same kinds of effects on developmental traits as for the standard diet where protein content was reduced by 40%, but expressed them to a greater degree. This suggests that despite the reduced growth rate on the novel diet, which might have allowed longer for resources to be accrued, the individuals surviving to pupariation could not maintain a stable adult phenotype, and paid a cost in terms of body size. The pattern seen in the novel carbohydrate treatment (see also [18]) may reflect the impact of novel carbohydrates on the metabolic control of development [13], or on the efficiency of metabolism during development [44], [45].

Considering responses to diet in the context of colonisation and invasion, responses to novel proteins may be less important than for other nutrients, as protein content of host fruit is generally low (0.86±0.59% [52] and invariant [53]. The carbohydrate content in fruits is, however, higher and more variable (13.7±13.7%; [52], [53]). The availability of carbohydrates will also vary across the range of hosts into which individuals may oviposit over the fruiting season, and also within hosts during the course of ripening and decay. The plasticity we observed is likely to be crucial in coping with such fluctuations and facilitating successful development. This is particularly so when considering the role that factors such as fruit structure [54] and secondary metabolites [55]–[57] may have within novel hosts.

Developmental plasticity reflects the ability of the Medlfy to adopt a range of nutritional rails [8] dependent upon the nutrients it encounters. Medflies exhibit behavioural adaptations to heterogeneous nutrients during development, such as larval migration to areas of higher nutritional quality within a host [58]. In dietary conditions that are nutritionally homogenous, these behavioural adaptations become obsolete. The results of this study are consistent with the hypothesis that the Medfly exhibits developmental plasticity, manifested as the ability to travel down different nutritional rails. Unsurprisingly, less efficient and less successful nutritional rails are the only options to follow when the nutritional space comprises novel components. We suggest that the application of methods designed to define and control intake of nutrients will be extremely useful and may offer insight into the apparently atypical responses of medfly lifespan to dietary restriction [59].

Overall, the results of this study highlight the potential flexibility of phytophagous insects such as the Medfly. The plasticity seen in developmental traits gives insight into the ability of this wide-ranging generalist to adapt to variation within its nutritional environment. The swift global radiation of the Medfly has presumably favoured the spread of alleles that facilitate developmental success in many nutritional environments. This is reflected in the alteration of survival and developmental duration, and the maintenance of a relatively stable adult body mass in those individuals that do survive. For a generalist species, this allows resilience to fluctuation in nutrient availability both within and across hosts. This adaptive ability has also fostered resilience to harsh nutritional environments and maximises fitness even in radically different, novel host environments.

## Supporting Information

Figure S1 **Rate of emergence for pupal formation and adult eclosion.** Points represent mean data, solid lines represent GLM ANCOVA models fitted with proportion of pupae/adults emerged as main effect and experimental day as a covariate. Shaded regions indicate 95% confidence interval of the model. Error bars represent 1 standard error. (a) Rate of pupal formation (number of pupae forming per day) in the protein experiment. (b) Rate of pupal formation (number of pupae forming per day) in the carbohydrate experiment. (c) Rate of adult eclosion (number of adults eclosing per day) in the protein experiment. (d) Rate of adult eclosion (number of adults eclosing per day) in the carbohydrate experiment.
(PDF)

Figure S2 **Mean pupal weight (µg) in relation to day of emergence. Points represent mean data; solid lines represent ANCOVA models fitted with mean pupal weight as a main effect and emergence day as a covariate.** Shaded regions indicate 95% confidence interval of the model. Error bars represent 1 standard error. (a) Protein experiment, (b) Carbohydrate experiment.
(PDF)

Table S1 **The proportion of Medfly larvae forming pupae per day when reared on four diets was examined using GLM ANCOVA.** Diet was the main effect and experimental day was a covariate. The table below describes the differences in intercept and gradient between the model lines fitted to each dietary treatment. Estimate values for lactose, maltose, and starch represent differences from the estimate values shown for glucose.
(DOCX)
