# Supplementary figures and images for: Effect of Dietary Components on Larval Life History Characteristics in the Medfly (Ceratitis capitata: Diptera, Tephritidae)
Source: PLoS One. 2014 Jan 21;9(1):e86029. doi: 10.1371/journal.pone.0086029 (PMC3897573; doi:10.1371/journal.pone.0086029)

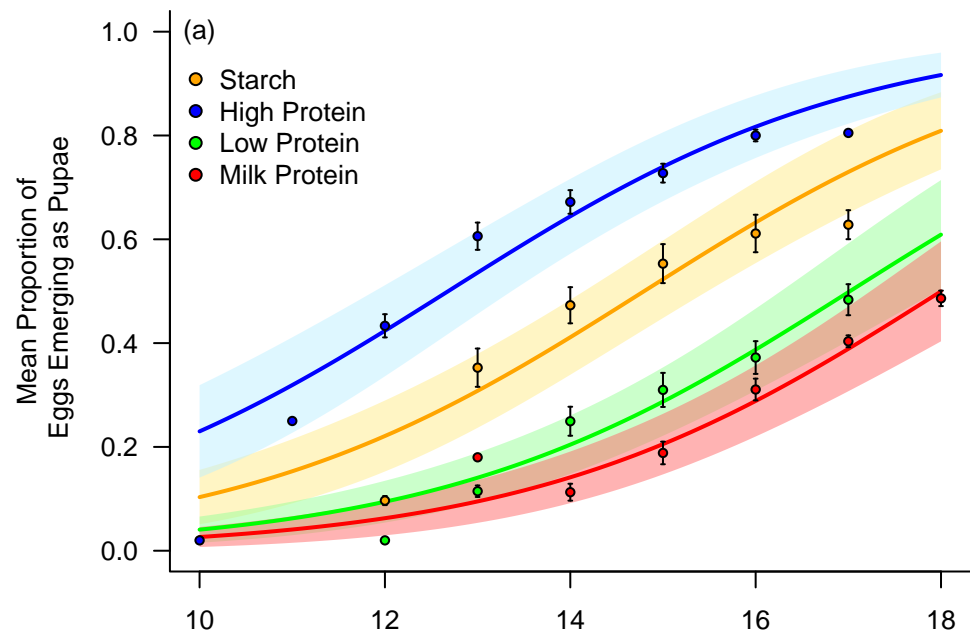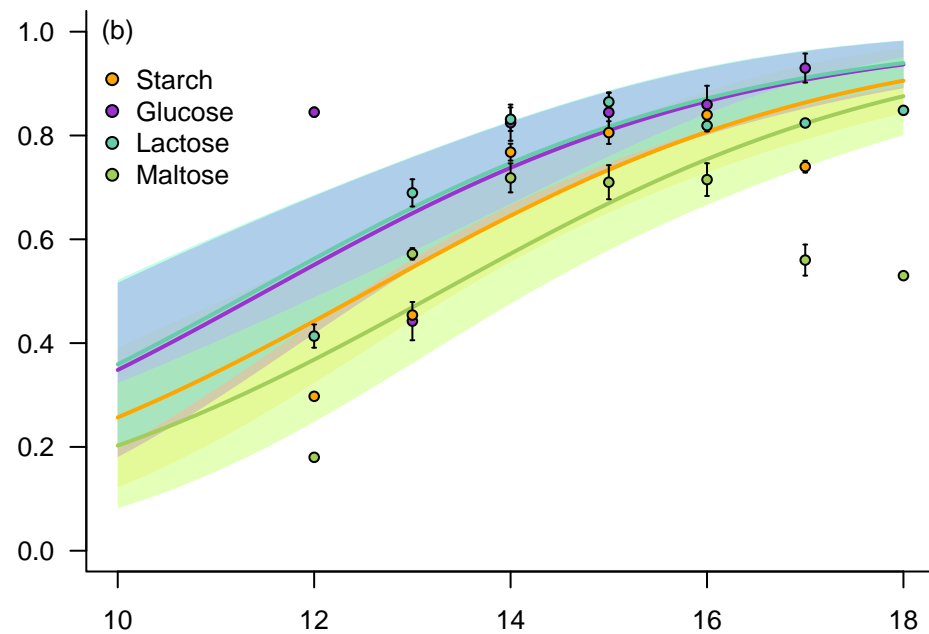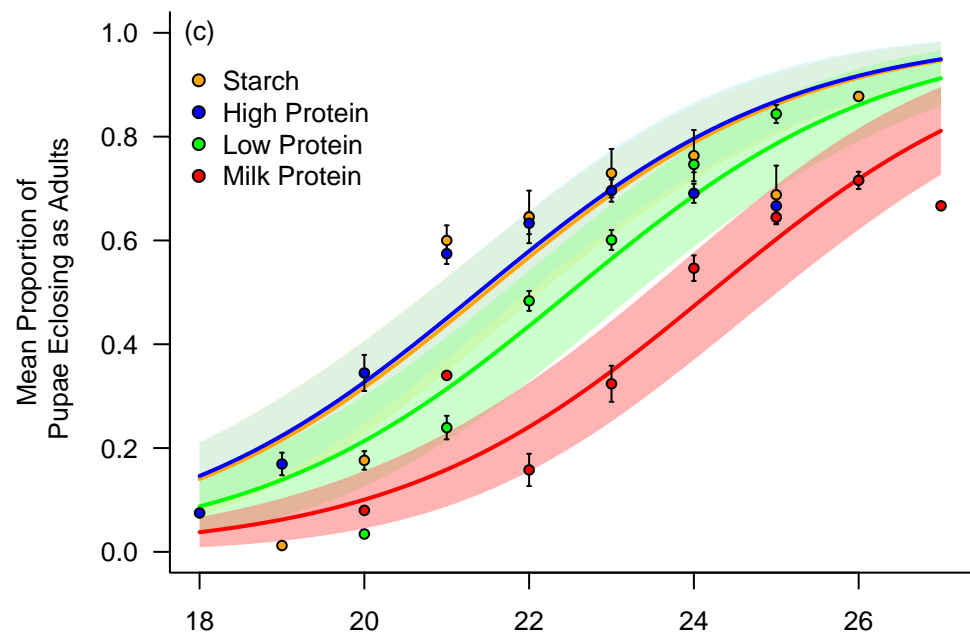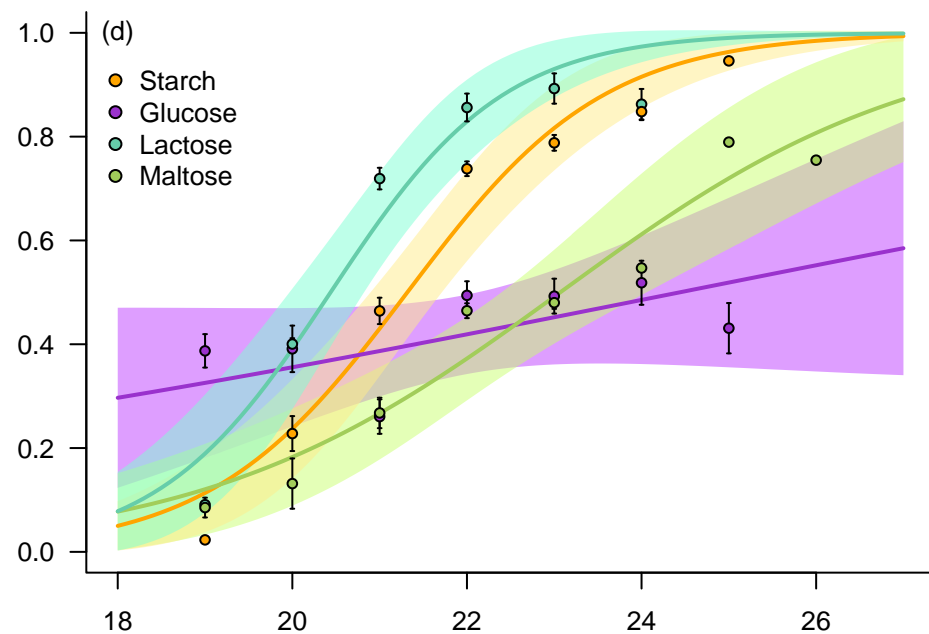

Experimental Day

Supplement: Figure S1 — Rate of emergence for pupal formation and adult eclosion. Points represent mean data, solid lines represent GLM ANCOVA models fitted with proportion of pupae/adults emerged as main effect and experimental day as a covariate. Shaded regions indicate 95% confidence interval of the model. Error bars represent 1 standard error. (a) Rate of pupal formation (number of pupae forming per day) in the protein experiment. (b) Rate of pupal formation (number of pupae forming per day) in the carbohydrate experiment. (c) Rate of adult eclosion (number of adults eclosing per day) in the protein experiment. (d) Rate of adult eclosion (number of adults eclosing per day) in the carbohydrate experiment. (PDF) [file pone.0086029.s001.pdf]

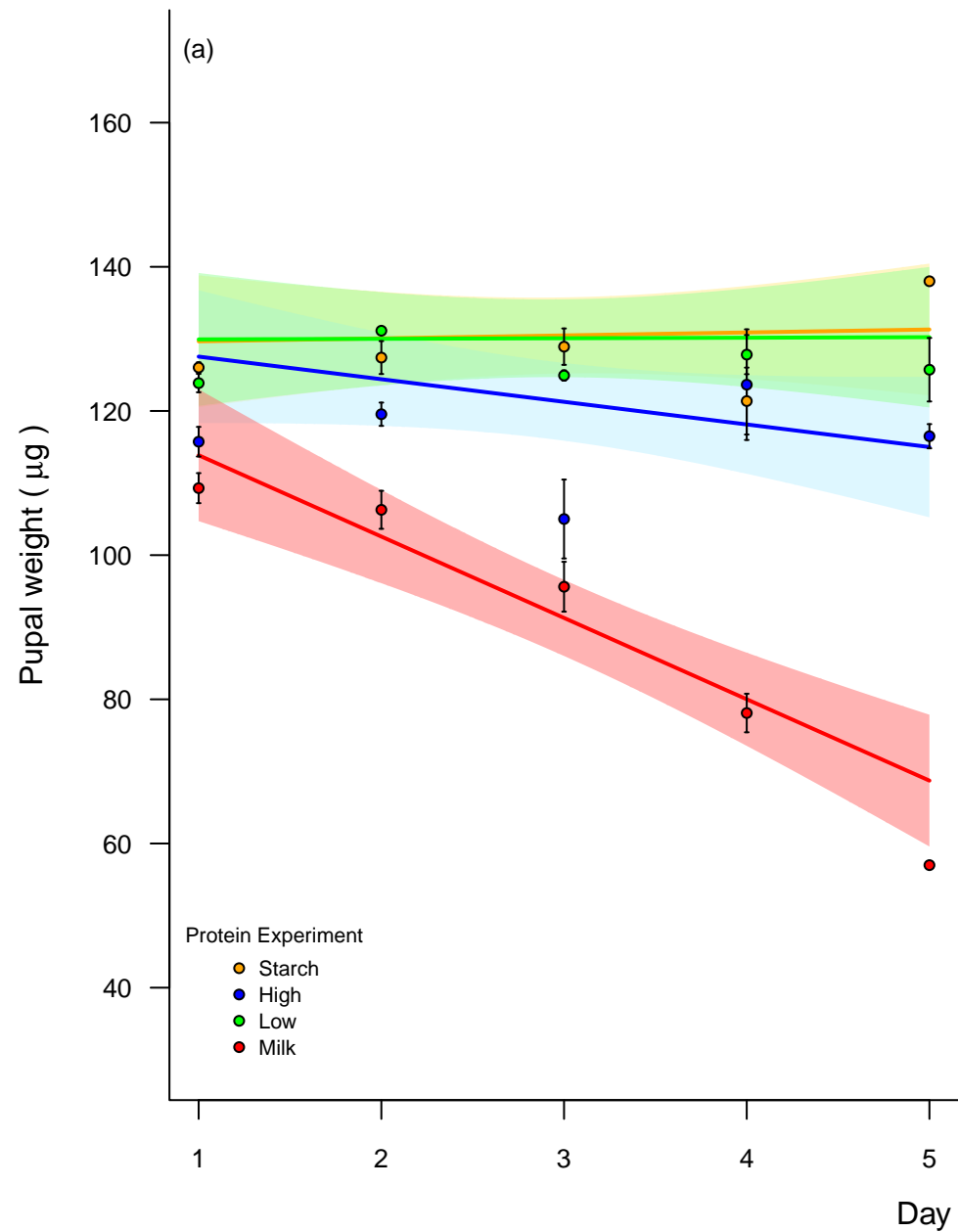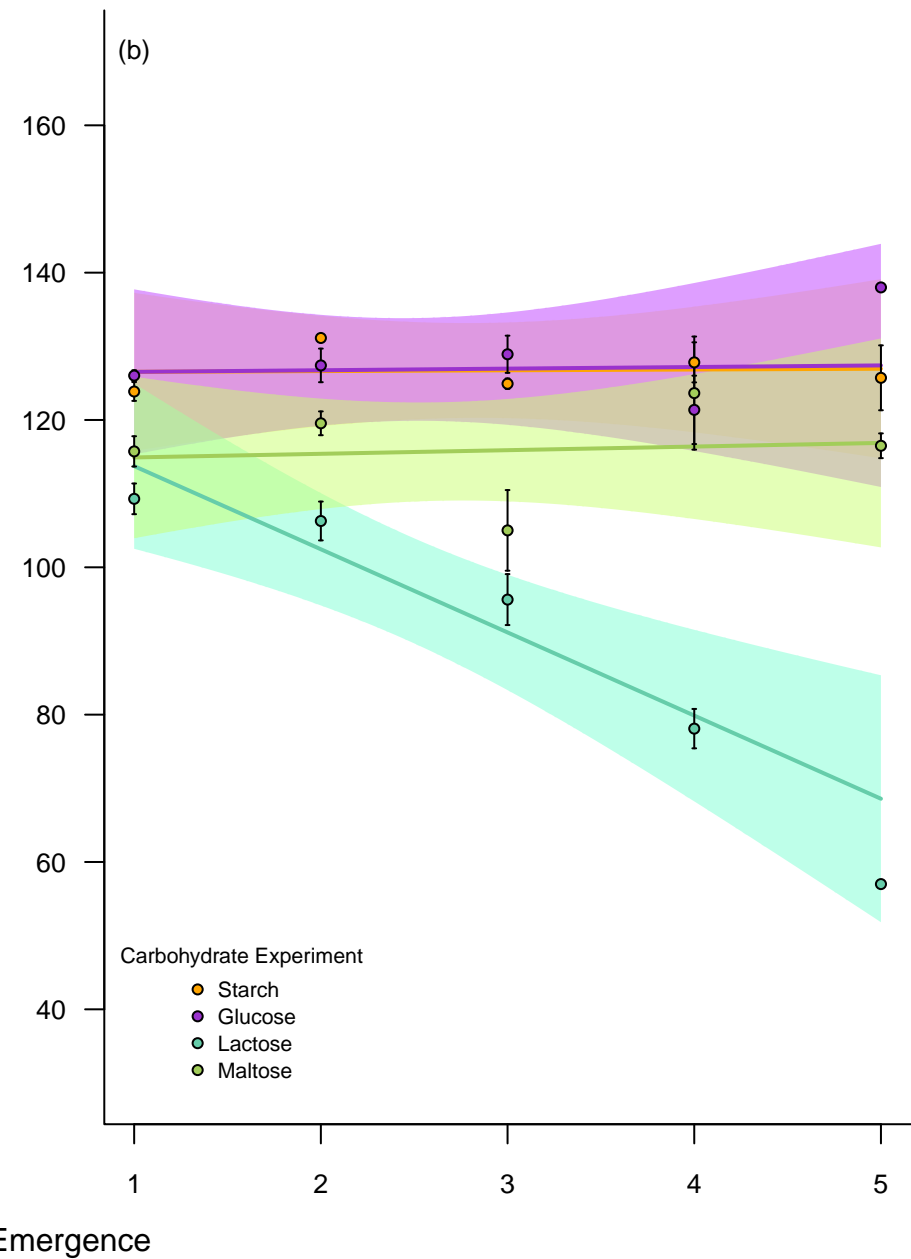

Supplement: Figure S2 — Mean pupal weight (µg) in relation to day of emergence. Points represent mean data; solid lines represent ANCOVA models fitted with mean pupal weight as a main effect and emergence day as a covariate. Shaded regions indicate 95% confidence interval of the model. Error bars represent 1 standard error. (a) Protein experiment, (b) Carbohydrate experiment. (PDF) [file pone.0086029.s002.pdf]
