# Supplementary material for: Effect of Dietary Components on Larval Life History Characteristics in the Medfly (Ceratitis capitata: Diptera, Tephritidae)
Source: PLoS One. 2014 Jan 21;9(1):e86029. doi: 10.1371/journal.pone.0086029 (PMC3897573; doi:10.1371/journal.pone.0086029)
Supplement: Table S1 — The proportion of Medfly larvae forming pupae per day when reared on four diets was examined using GLM ANCOVA. Diet was the main effect and experimental day was a covariate. The table below describes the differences in intercept and gradient between the model lines fitted to each dietary treatment. Estimate values for lactose, maltose, and starch represent differences from the estimate values shown for glucose. (DOCX) [file pone.0086029.s003.docx]

**Effect** **of dietary components on larval life history characteristics in the Medfly (*Ceratitis capitata*: Diptera, Tephritidae)**

W. J. Nash & T. Chapman

*School of Biological Sciences, University of East Anglia, Norwich Research Park, Norwich, Norfolk, UK.*

*Corresponding Author*: Will Nash, Email: w.nash@uea.ac.uk.

**Table S1**

The proportion of Medfly larvae forming pupae per day when reared on four diets was examined using GLM ANCOVA. Diet was the main effect and experimental day was a covariate. The table below describes the differences in intercept and gradient between the model lines fitted to each dietary treatment. Estimate values for lactose, maltose, and starch represent differences from the estimate values shown for glucose.

|  | Treatment | Estimate | Std. Error | t value | P value |
| --- | --- | --- | --- | --- | --- |
| Intercept | Glucose | -3.2732 | 2.16046 | -1.515 | 0.1328 |
|  | Lactose | -17.4003 | 4.25614 | -4.088 | < 0.001 |
|  | Maltose | -7.99042 | 3.34455 | -2.389 | 0.0187 |
|  | Starch | -15.6391 | 3.66741 | -4.264 | < 0.001 |
| Gradient | Glucose | 0.13395 | 0.09789 | 1.368 | 0.1742 |
|  | Lactose | 0.87758 | 0.20127 | 4.36 | < 0.001 |
|  | Maltose | 0.35432 | 0.15013 | 2.36 | 0.0202 |
|  | Starch | 0.75324 | 0.16906 | 4.455 | < 0.001 |
